# Supplementary material for: Transcriptomic Insights into Mechanisms of Early Seed Maturation in the Garden Pea (Pisum sativum L.)
Source: Cells. 2020 Mar 23;9(3):779. doi: 10.3390/cells9030779 (PMC7140803; doi:10.3390/cells9030779)
Supplement: Supplementary file 1 [file cells-09-00779-s001.zip › Supplementary materials/FileS3.zip!/FileS2/Hormones - Results/Summary Sheets - Differences/0verview.html]

Differences Summary of Hormones dataset


# Differences Analyses

## Pairwise Difference Summary Sheets

For each pairwise comparison a report sheet, gene lists and gene set
lists are created analogous to the Sample Summary Sheets.

| Comparison | Summary Sheet | Global Gene List | Gene Set List |
| --- | --- | --- | --- |
| Sprint-2(10) vs Sprint-2(20) | PDF | CSV | CSV |
| Sprint-2(10) vs Zhewan-1(10) | PDF | CSV | CSV |
| Sprint-2(10) vs Zhewan-1(25) | PDF | CSV | CSV |
| Sprint-2(10) vs Zhongwan-6(10) | PDF | CSV | CSV |
| Sprint-2(10) vs Zhongwan-6(25) | PDF | CSV | CSV |
| Sprint-2(20) vs Zhewan-1(10) | PDF | CSV | CSV |
| Sprint-2(20) vs Zhewan-1(25) | PDF | CSV | CSV |
| Sprint-2(20) vs Zhongwan-6(10) | PDF | CSV | CSV |
| Sprint-2(20) vs Zhongwan-6(25) | PDF | CSV | CSV |
| Zhewan-1(10) vs Zhewan-1(25) | PDF | CSV | CSV |
| Zhewan-1(10) vs Zhongwan-6(10) | PDF | CSV | CSV |
| Zhewan-1(10) vs Zhongwan-6(25) | PDF | CSV | CSV |
| Zhewan-1(25) vs Zhongwan-6(10) | PDF | CSV | CSV |
| Zhewan-1(25) vs Zhongwan-6(25) | PDF | CSV | CSV |
| Zhongwan-6(10) vs Zhongwan-6(25) | PDF | CSV | CSV |
